# Comparative genomics reveals insights into anuran genome size evolution

**DOI:** 10.1186/s12864-023-09499-8

**Published:** 2023-07-06

**Authors:** Bin Zuo, Lotanna Micah Nneji, Yan-Bo Sun

**Affiliations:** 1 Ministry of Education Key Laboratory for Transboundary Ecosecurity of Southwest China, Yunnan Key Laboratory of Plant Reproductive Adaptation and Evolutionary Ecology, Institute of Biodiversity, School of Ecology and Environmental Science, Yunnan University, Kunming, Yunnan 650504 China; 2 Department of Ecology and Evolutionary Biology, Princeton University, Princeton, NJ 08544 USA; 3 Laboratory for Conservation and Utilization of Bio-resources, Yunnan University, Kunming, 650091 China

**Keywords:** Genome size variation, Simple repeat sequences, Anurans, Transposable elements

## Abstract

**Background:**

Amphibians, particularly anurans, display an enormous variation in genome size. Due to the unavailability of whole genome datasets in the past, the genomic elements and evolutionary causes of anuran genome size variation are poorly understood. To address this, we analyzed whole-genome sequences of 14 anuran species ranging in size from 1.1 to 6.8 Gb. By annotating multiple genomic elements, we investigated the genomic correlates of anuran genome size variation and further examined whether the genome size relates to habitat types.

**Results:**

Our results showed that intron expansions or contraction and Transposable Elements (TEs) diversity do not contribute significantly to genome size variation. However, the recent accumulation of transposable elements (TEs) and the lack of deletion of ancient TEs primarily accounted for the evolution of anuran genome sizes. Our study showed that the abundance and density of simple repeat sequences positively correlate with genome size. Ancestral state reconstruction revealed that genome size exhibits a taxon-specific pattern of evolution, with families Bufonidae and Pipidae experiencing extreme genome expansion and contraction events, respectively. Our result showed no relationship between genome size and habitat types, although large genome-sized species are predominantly found in humid habitats.

**Conclusions:**

Overall, our study identified the genomic element and their evolutionary dynamics accounting for anuran genome size variation, thus paving a path to a greater understanding of the size evolution of the genome in amphibians.

**Supplementary Information:**

The online version contains supplementary material available at 10.1186/s12864-023-09499-8.

## Background

Eukaryotic genomes vary greatly in size, with existing genomes differing in size by about 200,000-fold, thus providing an open question about the evolutionary forces that shape the genome size evolution [1]. Several studies have conducted comparative genomic analyses of closely related and allopatric species to understand the evolutionary factors underlying genome size variation across multiple taxa [2–6]. One popular explanation is that the proliferation and contraction of repetitive sequences play an essential role in the evolution of eukaryotic genome size [4, 7–11]. Moreover, gene or genome duplication [12–15] and intron size changes [10, 16, 17] have also been implicated as underlying evolutionary forces causing variations in genome size. Further, a significant correlation between genome sizes and the abundance and density of simple sequence repeats (SSRs) was also reported [18, 19]. Unfortunately, the comprehensive characteristics and dynamics underlying eukaryotic genome size evolution remain contentious.

Repetitive sequences are abundantly distributed in eukaryotic genomes and can influence genome size. It can be categorized based on their sequence features or the process by which they are created. One major category consists of transposable elements (TEs), which include sequences that are dispersed throughout the entire genome and can move and multiply across the host genome [20]. The TEs are further divided into two groups [21–24], with Class I transposons (retrotransposons) which make new copies using an RNA-mediated copy-and-paste mechanism [25], and Class II transposable elements (DNA transposons) which replicate elements using a DNA-mediated cut-and-paste mechanism [26]. Studies have shown that retrotransposons occupy a significant fraction of TEs in the eukaryotic genome [11, 27]. Based on the structural features, retrotransposons are further subdivided into Long Terminal Repeat retrotransposons (LTRs), Long Interspersed Nuclear Elements (LINEs) and Short Interspersed Nuclear Elements (SINEs). Another category of repetitive sequences is the tandem repeats, which include sequences found in consecutive copies along the DNA. This group comprises minisatellites and microsatellites or simple sequence repeats (SSRs) [28, 29]. The SSRs comprise Perfect SSRs (P-SSRs), Compound SSRs (C-SSRs), and Imperfect SSRs (I-SSRs) and are distributed in eukaryotic genomes [30]. Although some studies have shown that SSRs occupy only about 1% of the genome [31, 32], others reported that SSRs occupy up to 23% of the genome [33].

Anurans (collective term for frogs and toads) are a suitable model for investigating mechanisms underlying divergence in genome size owing to their diverse genome sizes [34, 35]. With the advent of advanced sequencing technology, genomes of several anurans are now available, thus contributing ideal resources for exploring the characteristics and dynamics of genome size evolution. Although the dynamic evolution of repetitive sequences significantly contributes to eukaryotic genome size evolution, the relative contributions of these sequences to genome size evolution in anurans remained controversial [36, 37]. For instance, a recent study on cave frog (*Platyplectrum ornatum*) suggested that reduction of intron abundance, loss of TEs, and suppression of activity could be the primary causes of genome contraction [16]. Also, Wang et al. (2021) reported no significant relationship between TEs diversity and genome size [38]. In contrast, Haley and Mueller (2022) showed that larger genome size was associated with increased TE diversity at the superfamily level [39]. Hence, these previous studies failed to obtain consistent results on the genomic elements contributing to anuran genome size evolution. Additionally, anurans occupy diverse habitats (e.g., forests, savanna, humid ecosystems etc.) and migrate between habitats for breeding. Yet, it is unknown if anurans modify their genome size due to species-specific habitat requirements and life cycles [40, 41].

Here, we conducted comparative genomic analyses of fourteen anurans that include species ranging in genome size from *Spea multiplicata* (1.1GB) to *Ranitomeya imitator* (6.8GB) to understand the mechanism(s) underlying genome size evolution. Specifically, we seek to answer the following questions: (1) What are the exact genomic elements (TEs, SSR or both) accounting for the genome size variation in anurans? (2) How do non-coding regions (intron, exon, and intergenic regions) drive anuran genome size evolution? (3) What are the evolutionary causes of the genome size evolution in anurans? And (4) Does the anuran genome size correlate with habitat types? The results of our study offer deep insights into the genomic elements and evolutionary mechanisms underlying the evolution of anuran genome size.

## Results

### Repetitive sequence landscape

**Fig. 1:**
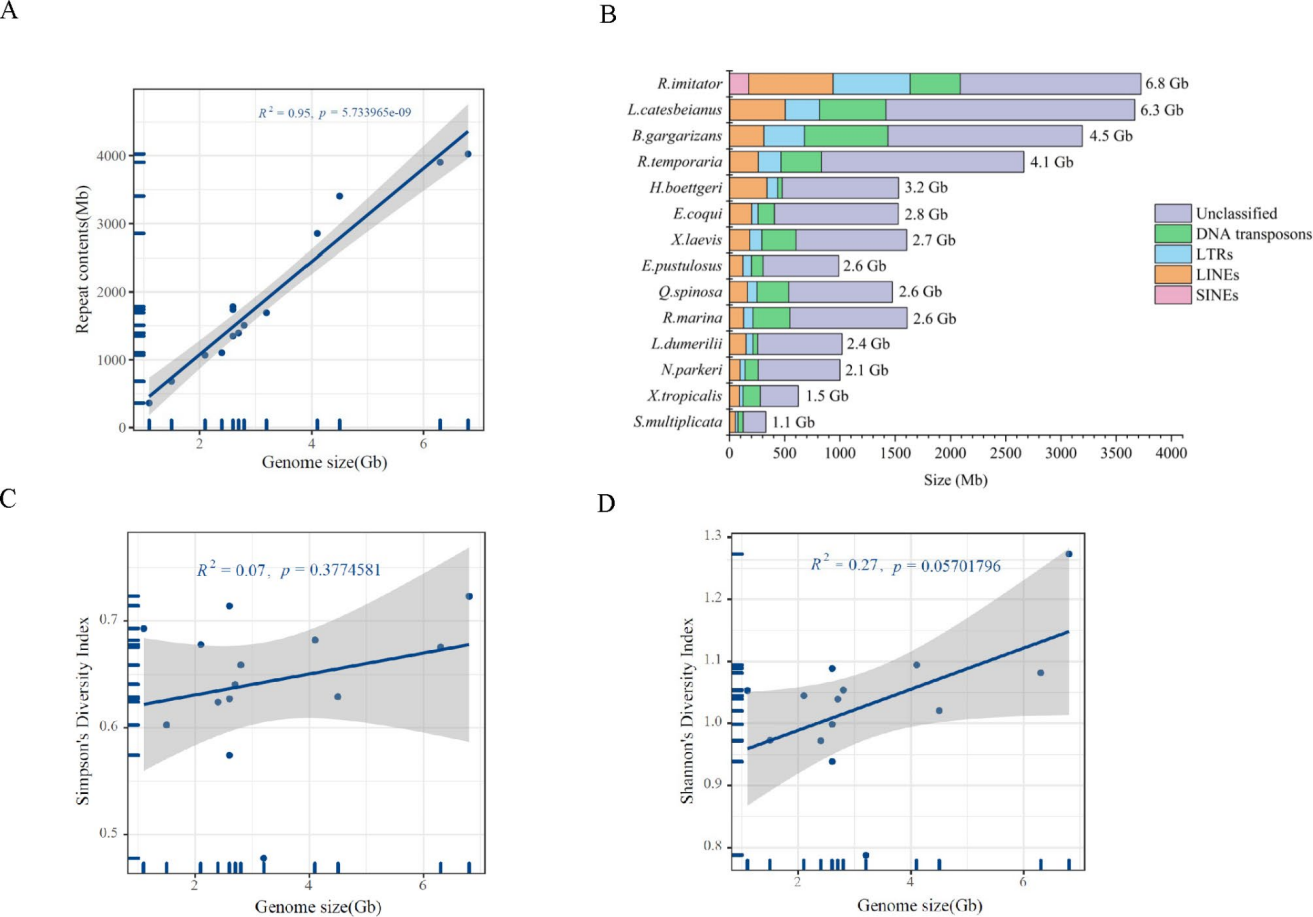
Shows the association between genome size and repetitive sequences/transposable elements diversity index in anuran species. **(A)** Positive correlation between genome size and repetitive elements. The X-axis shows the genome size, the length of the repetitive elements by the Y-axis, and the 95% confidence interval by the gray region. **(B)** Abundance and Distribution of the Transposable elements across anuran species. **(C)** Genome size and transposable element diversity index (Simpson diversity index). **(D)** Genome size and transposable element diversity index (Shannon Diversity Index). Gray shade represents the 95% confidence interval

Repetitive sequences were more abundant in species with larger genome sizes (Supplementary Materials 1: Table S3). Furthermore, there was a significant correlation (*R*^2^ = 0.95, *P* = 5.733965e^-09^) (Fig. 1A) between the genome size and abundance of all repetitive sequences based on the result of the combined analysis using RepeatModeler, RepeatMasker, and TRF. Analyses showed that *R. imitator* (genome size = 6.8GB) and *L. catesbeianus* (genome size = 6.3GB) had higher repetitive sequences of 59.3% and 62.5%, respectively. However, only 33.7% of repetitive sequences were found in the *S. multiplicata* (genome size = 1.1GB).

We observed a species-specific association of the TEs abundance with the genome size. For instance, while *S. multiplicata* (genome size = 1.1 Gb) exhibited a persistent reduction in multiple repetitive sequence classes (Fig. 1B), we found multiple repetitive sequences within the genomes of *L. catesbeianus* (genome size = 6.3 Gb) and *R. imitator* (genome size = 6.8 Gb). The larger genomes contain a higher proportion of repetitive sequences, suggesting the proliferation of repetitive sequences as the leading cause of genome expansion (Fig. 1B). Besides, analyses showed that although DNA transposons, LINEs and LTRs occupied a large proportion of the anuran genome, different correlations with genome size were observed (Supplementary Materials 2: Fig. S3; Supplementary Materials 1: Table S4). The linear regression model showed that LINEs had the strongest positive correlation (*R*^2^ = 0.89, *P* = 4.01e^-07^) with genome size, followed by LTRs (*R*^*2*^ *= 0.82, P =* 8.54e^-06^), DNA transposons (*R*^2^ = 0.55, *P* = 2.56e^-03^) and SINEs (*R*^2^ = 0.40, *P* = 1.53e^-02^). Except for *R. imitator* (SINE = 2.69%), the proportion of SINEs was extremely low (< 0.3%) in all species. This result suggests that SINEs have little impact on anuran genome size variation (Fig. 1B; Supplementary Materials 1: Table S4).

Based on the identification of tandem repeat sequences in the genomes of 14 species, our results reveal that satellite DNA constitutes a proportion ranging from 0.0022 to 0.43% (63,538 − 6,275,648 bp) of the genome. At the same time, SSRs account for a proportion ranging from 0.47 to 2.11% (21,272,043 − 67,891,025 bp) (Supplementary Materials 1: Table S8). Additionally, we found no correlation between genome size and the total length (*R*^2^ = 0, *P* = 0.9001969) and number (*R*^2^ = 0.14, *P* = 0.1945149) of satellite sequences (Supplementary Materials 2: Fig. S6). However, a significant correlation was observed between genome size and the total length (*R*^2^ = 0.55, *P* = 0.002242437) and number (*R*^2^ = 0.71, *P* = 0.0001458194) of simple repeats (Supplementary Materials 2: Fig. S6).

### TEs community diversity and dynamics

We observed no strong positive correlation between genome size and TEs diversity (Simpson Diversity Index *R*^2^ = 0.07, *P* = 0.3774581, Shannon Diversity Index *R*^2^ = 0.27 *P* = 0.05701796) (Fig. 1C and D; Supplementary Materials 1: Table S2). However, a weak positive correlation between the Shannon diversity index of TEs and genome size was seen.

**Fig. 2:**
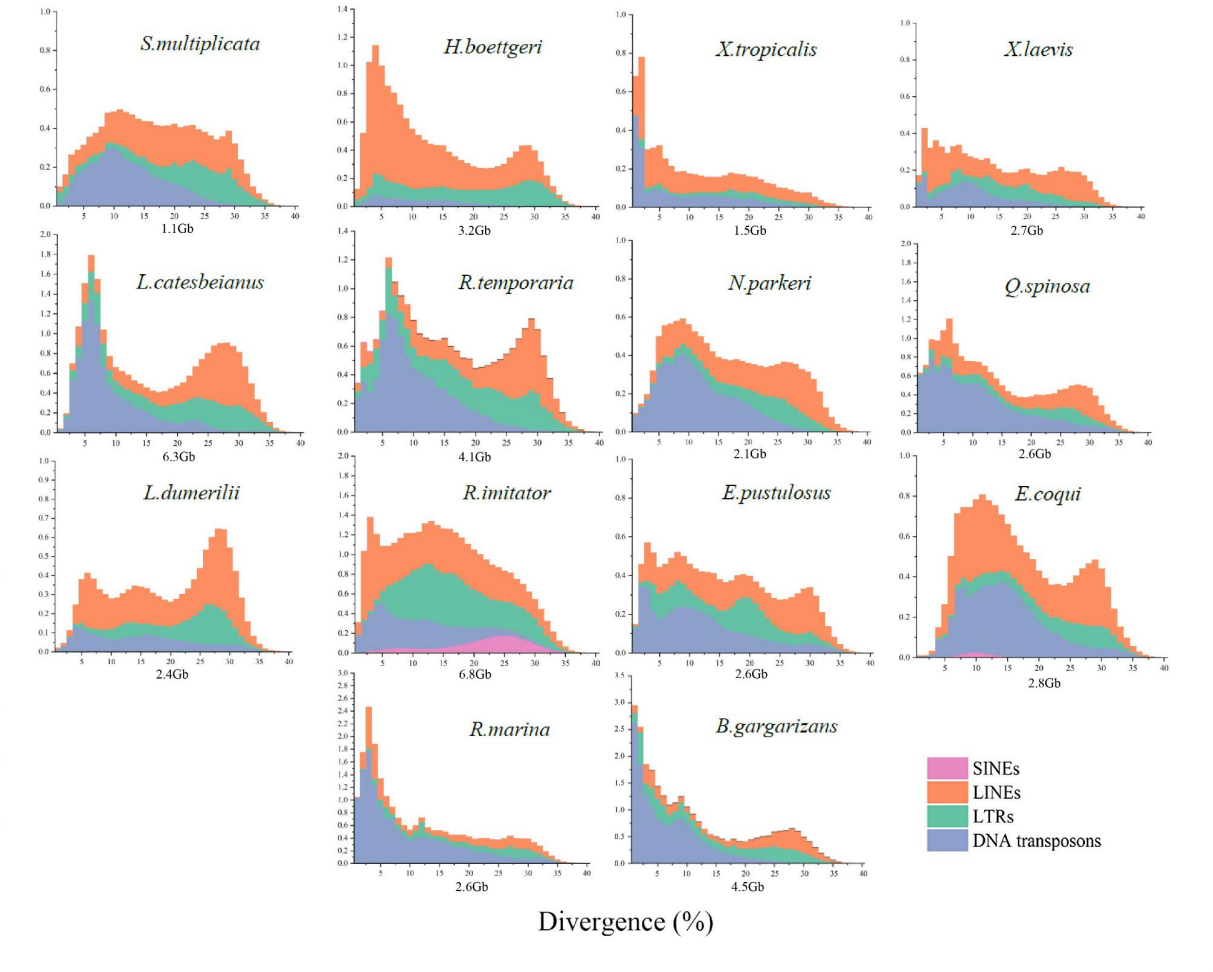
Transposable element age distribution landscapes of anuran genome sizes. The Y-axis shows the genomic coverage of different types of TEs, and the X-axis shows the Kimura substitution level as a percentage from 0 to 40. The Y-axis represents TE abundance as a proportion of the genome (e.g., 1.0 = 1% of the genome). The distribution landscape of TE divergence is categorized as follows: L-shaped distribution (TE divergence peak less than or equal to 5%), bimodal distribution (two peaks occur), or multi-peaked distribution (more than two peaks occur)

The divergence curves showed an L-shaped distribution for *X. tropicalis* and *R. marina* with a divergence of below 5%, which suggests a recent burst event (Fig. 2; Supplementary Materials 2: Fig. S8). Other species, including *H. boettgeri, S. multiplicata, X. laevis, L. catesbeianus, R. temporaria, B. gargarizans, N. parkeri, Q. spinosa, L. dumerilii, R. imitator, E. coqui, and E. pustulosus*, exhibited a bimodal or multi-peaked distribution. In this case, there were two or more peaks, with the first peak occurring at approximately 0–10% divergence and the second or third peak occurring at about 30% divergence, indicating a recent accumulation of TEs and a lack of deletion of more ancient TEs (Fig. 2; Supplementary Materials 2: Fig. S8). Although we observed two or more burst patterns, our result showed that the accumulation and lack of deletion of more ancient TE activity are mainly responsible for anuran genome size variation.

### Intact LTR-RTs amplification

We observed species-specific amplification of intact LTR-RTs in anuran genomes, with different insertion abundances across species (Supplementary Materials 2: Fig. S4). Species with larger genome sizes, such as *L. catesbeianus* and *R. imitator*, had relatively low insertion abundances. In contrast, species with medium genome sizes, such as *B. gragraizans* and *R. temporaria*, had the highest insertion abundance (Supplementary Materials 2: Fig. S4). In particular, intact LTR-RTs in *B. gragraizans* displayed high abundance and a recent insertion abundance (< 1 MYA) compared to other species (Supplementary Materials 2: Fig. S4). Our results indicate that the large genome size is attributed to older LTR activity, which implies a higher probability that the LTR elements inserted earlier in the genome will not be intact.

### Microsatellite sequence landscape

**Fig. 3:**
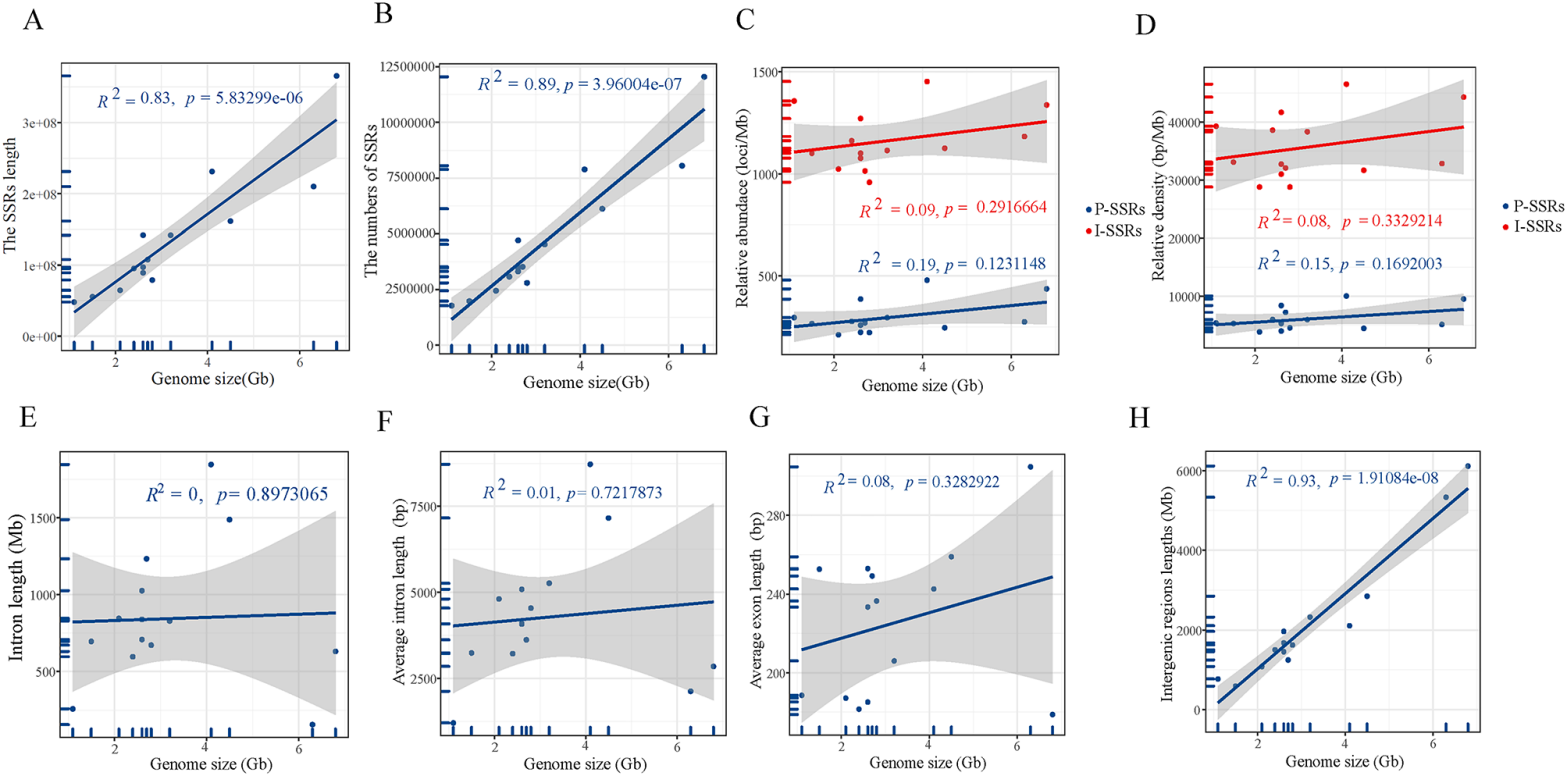
Relationship between anuran genome size and exons, introns, intergenic regions and Simple Sequence Repeats, respectively. (Gray shade represents the 95% confidence interval) Figures A, B, C, D, E, F, G, and H represent the relationship between genome size and length of SSRs, number of SSRs, relative abundance of SSRs, relative density of SSRs, intron length, average intron length, average exon length, and intergenic region length for 14 anuran species

The percentage of SSRs in anuran genomes ranges from approximately 2.84–5.63% (79,217,477 bp − 231,473,092 bp), with the highest rate (5.63%, 231,473,092 bp) found in the genome of *R. temporaria* and the lowest (2.84%, 79,217,477 bp) in *E. coqui* (Supplementary Materials 1: Table S5). Our analysis revealed a positive correlation between genome size and the total length and number of SSRs (*R*^2^ = 0.83 *P* = 5.83299e^− 06^, *R*^2^ = 0.89 *P* = 3.96004e^− 07^) (Fig. 3A and B). As an implication, the positive correlation between genome size and the total length and number of SSRs suggests that SSRs experienced expansion within the anuran genome and contributed to the genome size. Nevertheless, genome size did not correlate with the relative abundance (I-SSRs: *R*^2^ = 0.09 *P* = 0.2916664, P-SSRs: *R*^2^ = 0.19 *P* = 0.1231148) and relative density of SSRs (I-SSRs: *R*^2^ = 0.08 *P* = 0.3329214, P-SSRs: *R*^2^ = 0.15 *P* = 0.1692003) (Fig. 3C and D). We also observed that the six categories of SSRs had different abundances in I-SSRs and P-SSRs (Supplementary Materials 2: Fig. S2). Specifically, Mononucleotide, Dinucleotide and Tetranucleotide were the most abundant types of SSRs among the P-SSRs in the 14 anuran genomes (Supplementary Materials 2: Fig. S2B). In contrast, Mononucleotide, Dinucleotide and Trinucleotide were the three most abundant types of I-SSRs (Supplementary Materials 2: Fig. S2A). These results suggest that Mononucleotide, Dinucleotide, Trinucleotide, and Tetranucleotide are the main categories of SSRs that shape the genomes of the 14 anuran species.

### Distribution of the non-coding regions of the genome

We found no correlation between genome size and intron length (*R*^2^ = 0, *P* = 0.8973065) (Fig. 3E; Supplementary Materials 2: Fig. S1). To further verify this, we compared the average intron and exon lengths in the genomes. Similarly, genome size failed to correlate strongly with the average intron length (*R*^2^ = 0.01, *P* = 0.7217873) and average exon length (*R*^2^ = 0.08, *P* = 0.3282922) (Fig. 3F and G). Our result further showed an increase in the length of the intergenic region as the genome size increases (Fig. 3H; Supplementary Materials 2: Fig. S1B). Additionally, the exon abundance of the other 13 species was found to be lower when compared to *X. laevis* (Supplementary Materials 2: Fig. S1B).

### Phylogenetic estimation and ancestral state reconstruction

**Fig. 4:**
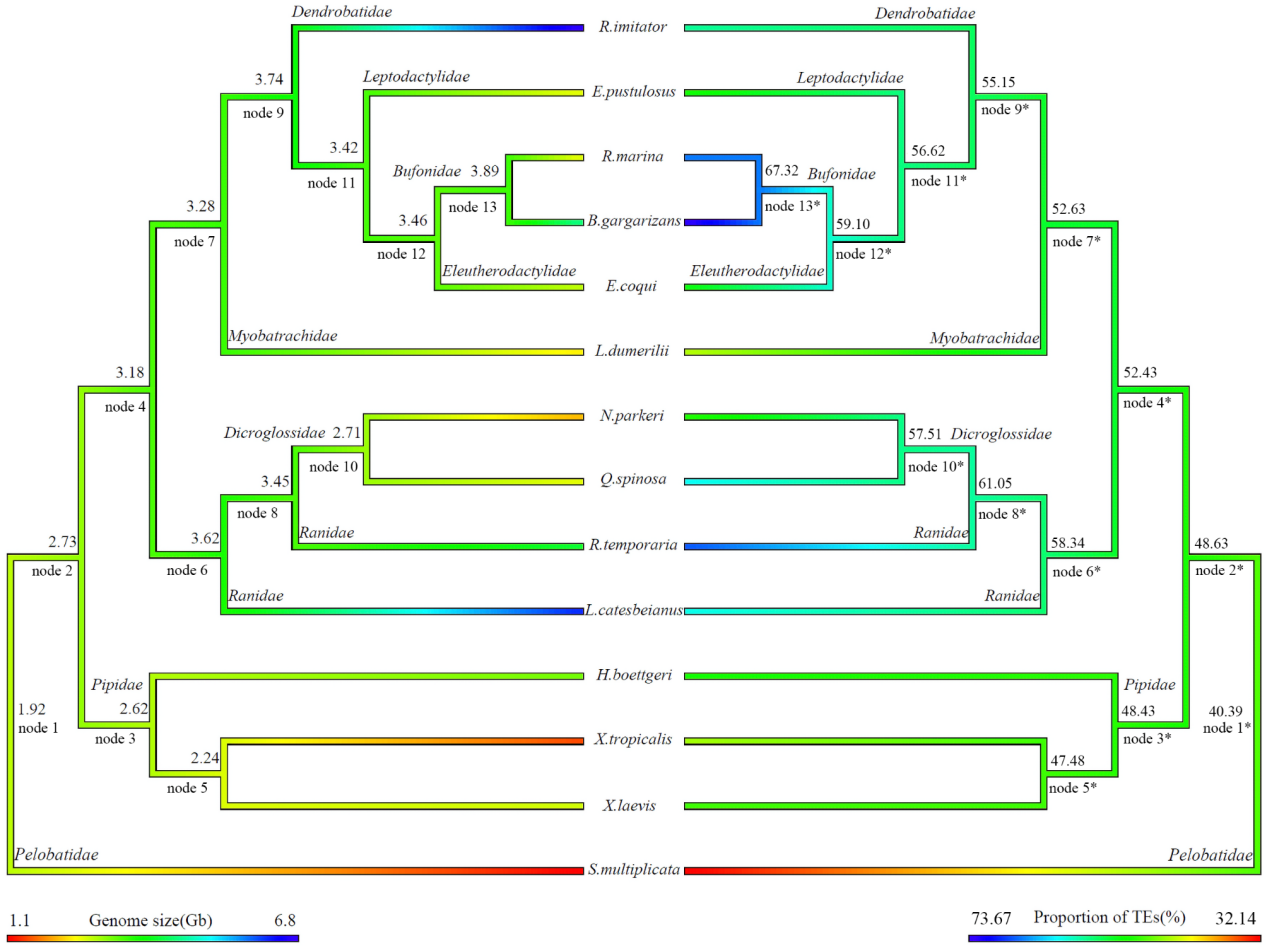
Reconstruction ancestral state of anuran genome size(left) and proportion of Transposable Elements(right) across 14 anuran species. Branching colors represent values reconstructed from phylogenetic relationships. The names and values on the nodes represent ancestral names and ancestral values for the branches, including Pipidae, Ranidae, Bufonidae, Dendrobatidae, Dicroglossidae, Eleutherodactylidae, Leptodactylidae, Myobatrachidae and Pelobatidae. In the figure, the nodes in the size branch of the genome (left) are represented by “node + number” (e.g., node 1), and the nodes in the TE branch are represented by “node + number*” (e.g., node 1*)

To understand the evolutionary relationship between anuran genome size and TEs abundance, we classified the evolutionary branches based on anuran families: Pipidae, Ranidae, Bufonidae, Dendrobatidae, Dicroglossidae, Eleutherodactylidae, Leptodactylidae, Myobatrachidae and Pelobatidae (Fig. 4). Our results indicate that the common ancestor of all species (node 1) had a small genome size (genome size = 1.92Gb) and a low TE abundance (node 1*) (proportion of TEs = 40.39%). However, we discovered that the ancestral genome size increased as the species diverged (Fig. 4). In the family Bufonidae, genome size tends to increase gradually as the species continued to diverge (node 9 to node 13) (although there were occasional intermediate cases of smaller genomes), reaching a peak at the ancestral nodes (Genome size = 3.89Gb) for some species (e.g., *R.marina* and *B.gargarizans*) with the highest TEs proportion (TEs = 67.32%). In contrast, the family with the smallest ancestral genome (e.g., Pipidae) experienced continuous genome contraction as species diverged (From node 2 to node 5) (genome size = 2.73Gb, 2.62Gb and 2.24Gb). However, for some species, e.g., *R. imitator* and *L. catesbeianus*, a sudden genome size increase following divergence from the ancestral nodes was observed (nodes 9 and 6, respectively). In contrast, in families Ranidae and Dicroglossidae, the TEs of ancestral nodes experienced a single event of expansion and contraction (node 6* through node 10*) (proportion of TEs = 58.34%, 61.05%, and 57.51%) (Fig. 4).

In addition, we observed the species with large genome sizes do not have the highest percentages of TEs in their ancestral nodes. For instance, although *R. imitator* and *L. catesbeianus* had the largest genome size compared to other species, we do not observe high TEs in their ancestral nodes (node 9* and node 6*, respectively) (Fig. 4). However, ancestral nodes (From node 2 to node 9) of other families (Pipidae, Ranidae, Bufonidae, Dendrobatidae, Dicroglossidae, Eleutherodactylidae, Leptodactylidae, Myobatrachidae and Pelobatidae) had moderately large genome sizes and TEs proportions (Fig. 4).

### Relationship between genome size and habitat types

Our results showed no correlation (*R*^2^ = 0, *P* = 0.8274032) between genome size and habitat types (Supplementary Materials 2: Fig. S5). However, we observed that species with genome sizes ranging from 2.6-4.5Gb had the highest ecological niche width or ecological tolerance (Supplementary Materials 2: Fig. S5; Supplementary Materials 1: Table S6). Additionally, large genome-sized species (e.g., *L. catesbeianus* and *R. imitator*) tend to inhabit humid habitats. In contrast, species with smaller genomes (e.g., *S. multiplicata*) prefer more arid habitats (Supplementary Materials 1: Table S7).

## Discussion

In this study, we performed comparative analyses of 14 anuran genomes to systematically explore the mechanisms underlying genome size evolution. Our result was consistent with an earlier study, which suggested that TEs amplification drives the expansion of genome size and that genome size is positively correlated with TEs numbers [6]. Our result further showed a strong positive correlation between the quantity of SSRs and the anuran genome size. Although previous studies based on a few available genomes indicated that SSRs occupy only 1.00% of the genome in most species [31], our result showed that the SSRs occupy ~ 2.84–5.63% (79,217,477 − 231,473,092 bp) of the entire anuran genomes. This observation is consistent with an earlier study in penaeid shrimp [33], which reported SSR proportions higher than 1%, and the roles SSR plays in genome plasticity and adaptive evolution. It is apparent that SSRs underwent expansion within the anuran genome, contributing to genome size evolution. However, the evolutionary triggers of increased SSRs in anurans with larger genome sizes are unknown, and additional research on this may provide further insight.

Our results showed no correlation between intron length and anuran genome size. This result was not in tandem with previous studies highlighting intron reduction as one of the leading causes of reduced genome size in vertebrates, including frogs [16, 42]. The introns in vertebrate genomes are influenced by a variety of factors, such as life history [43], metabolic rate [44] and life span [45], which in turn may affect anuran genome size. Also, our result showed no correlation of the intron or exon size with the genome size, except for the intergenic regions that showed a strong positive association with the anuran genome size. In addition, some species with less than 3 Gb of genomes (e.g., *X. laevis*, *X. tropicalis*, *R. marina*, and *N. parkeri*, etc.) had a 1:1 ratio of introns to intergenic regions. Previous studies have suggested that genome size changes are in a balance between insertions and deletions [46] and that the same ratio of introns to intergenic regions may be due to TE insertions, where the rate of intron amplification is the same as that of intergenic regions [47]. Nevertheless, further research integrating multiple genomic tools is required to explicitly understand the effects of the non-coding region on the genome size evolution across diverse vertebrates.

Our study showed species-specific intact LTR-RTs amplification in the anuran genome. The reduction in LTR-RTs length is mainly induced by a continuous decrease in the number of intact LTR-RTs [48]. However, our results suggest that intact LTRs are rare in large genome-sized species, and their total LTR-RTs length is not reduced, perhaps due to the accumulation of unpaired LTRs (solo-LTRs and truncated LTR-RTs). In addition, we found that the expansion of TEs in the anuran genome is species-specific, and species may have produced different genome expansion patterns after diverging from a common ancestor. Although an early study found that TEs expansion events occurred within lineage rather than from a few shared ancestral bursts [8], a recent study observed that the expansion history of TEs existed both in ancestral burst events and in recent transposition bursts [49]. Based on TEs expansion history or TEs age analysis, our study showed that the persistent accumulation of TEs with two or more burst events was the primary factor determining the size of the anuran genome. These results suggest that the effect of amplification or contraction of TEs on the size of the anuran genome is mainly a continuous process.

Our study revealed that the common ancestor of all the studied species had a small genome and a low level of TEs proportion and further experienced a Brownian motion (jump in genome size) occurring after divergence to acquire a larger genome size [50]. The pattern of change in genome size and TEs appear taxon-specific. Our study showed a disparity in the proportions of TEs states in species and their ancestors. For instance, large genome-sized species (e.g., *R. imitator* and *L. catesbeianus*) and their ancestors had the lowest percentage of TEs state, with smaller genome-sized species (e.g., *R. marina* and *B. gargarizans*) and their ancestors having the most abundant TEs proportions. Recent studies suggest that low levels of piRNA silencing mechanisms may reduce the inhibition of TEs, leading to a burst of TEs [51]. We predict that the rate and accumulation of TEs expansion among the anuran genomes are closely related to the silencing mechanism of piRNAs; however, this requires further study.

Our results indicated no relationship between anuran genome size and habitat types, although large genome-sized species preferred humid habitats, whereas small genome-sized species frequently occur in arid habitats. Previous studies have shown that cell size and replication rates are influenced by genome size, leading to longer developmental times in species with larger genomes [40, 52–54]. For instance, spadefoot toads with smaller genomes exhibit significantly shorter development times. Subsequent studies have revealed a correlation between anuran habitats and their life history traits [50]. Factors such as temperature and high drought conditions can impact developmental cycles. Moreover, the genome size of anurans is closely linked to their developmental rate, with species possessing smaller genomes exhibiting shorter development periods [55, 56]. This can be attributed to the need for organisms under water shortage stress to complete their development quickly. The results we observed in this study are consistent with their conclusions [50].

In addition, previous studies have demonstrated a correlation between genome size and ecological breadth, whereby species with larger genomes tend to occupy a broader range within their ecological niche [57]. However, our analysis cannot test the hypothesis that ecological breadth contributes to genome size variation in anurans. Therefore, further studies are required to investigate the extent to which the ecological breadth of anurans can influence their genome size.

### Limitations of this study

Although this study provides valuable insights into the molecular mechanisms underlying anuran genome size evolution, certain limitations should be noted. First, the quality of genome assembly can influence the estimation of TE content and abundance. While most of the 14 genomes were assembled at the chromosomal level with genomic completeness exceeding 90.0%, there are a few species, such as *E. pustulosus*, *L. catesbeianus*, and *E. coqui*, with relatively lower assembly quality (completeness of only 75.7%, 46.5%, and 76.4%, respectively) perhaps due to technological limitations as at the time of genome assembly [58]. Analyses showed that the anuran genome sizes used in our study do not negatively impact the genome completeness (Supplementary Materials 2: Fig. S7). Secondly, different evaluation methods or technical approaches used in genome assembly can lead to inconsistent genome size estimation. For example, in previous studies, the genome size of *R. marina* was assessed using either densitometry or flow cytometry analysis of stained nuclei in erythrocytes, hepatocytes and renal cells, resulting in a range of estimates from 3.98 to 5.65 Gb [59–66]. However, more recent studies on *R. marina* used short-read k-mer distributions and quantitative PCR (qPCR) of single-copy genes to evaluate the genome size resulting in size estimates ranging from 1.98 to 2.38 Gb [67]. These discrepancies highlight that the variability in genome size estimation could depend on the chosen methodology of genome assembly, thus underscoring the importance of careful consideration and validation when assessing genome size. While we took several steps to include high-quality genome assembly in our study and the robustness of our analyses, the patterns revealed in our study may, to some extent, reflect the genomic and ecological correlates of anuran genome size variation. This study advances our understanding of amphibian genome size evolution and sets the stage for further investigations.

Additionally, there may be some limitations in studying SSRs using whole-genome approaches. The low quality of a genome, such as the presence of gaps or assembly errors, can potentially result in the loss of simple sequence repeats (SSRs). Past studies have indicated that SSRs are present in both protein-coding and non-coding regions of eukaryotic genomes [68, 69]. Therefore, assembly errors or the presence of gaps can decrease the detection rate of SSRs. Based on the results presented above, although three species in this study had low genome assembly quality, the genomes of the remaining 11 species exhibited good assembly quality. Therefore, this study can provide a reliable conclusion. The identification and profiling of repeats can be affected by sequencing errors, misalignments, or insufficient coverage, leading to the potential misidentification of repetitive sequences by the algorithms [70–74]. The absence of haplotype information in genome assemblies poses a challenge in distinguishing between simple repeat polymorphisms occurring within haplotypes and between haplotypes. Studying simple repeats using genome assemblies instead of raw reads can yield valuable insights into repeat evolution and function. However, it is important to exercise caution when interpreting the results, considering the possible presence of assembly errors and incomplete representation.

## Methods

### Genome retrieval and quality assessment

We obtained published whole genome assemblies of anurans from the National Center of Biotechnology and Information, DRYAD, gigaDB and GSA databases (Table 1). In the case of NCBI genome assemblies, we searched using the taxonomy database (keywords “anurans” and “amphibians”). In sum, we retrieved whole genomes of 14 anuran species (*Bufo gargarizans*, *Eleutherodactylus coqui, Engystomops pustulosus, Hymenochirus boettgeri*, *Limnodynastes dumerilii, Lithobates catesbeianus, Nanorana parkeri, Rana temporaria*, *Xenopus laevis, Xenopus tropicalis, Quasipaa spinosa, Rhinella marina, Spea multiplicata*, and *Ranitomeya imitator*) belonging to nine families. Genome-wide data versions, release dates and database information are available in the supplementary material (Supplementary Materials 1: Table S1). To ensure the accuracy of our datasets, following the methods of Ibrahim et al. [75], we collected the information on the genome completeness data of 14 anuran species from the NCBI and relevant citations (referring to Benchmarking Universal Single-Copy Orthologs (BUSCO) assessment results [76]). This data includes information such as the number of scaffolds, scaffold N50, contigs, contig N50, genome assembly level, and genome size (referring to the assembly size) (Supplementary Materials 1: Table S1). For species without BUSCO assessment results, e.g., *Lithobates catesbeianus*, we conducted genome completeness using BUSCO v5.4.4 [76] based on the vertebrata_odb10 (Version 2021-02-19) database. Finally, we assessed if the genome size could negatively impact the genome completeness.

**Table 1:** Sample information, including genome size and data sources

Species	Genome size	References	Accession number	
*Bufo gargarizans*	4.5	[96]		GCF_014858855.1
*Eleutherodactylus coqui*	2.8	[97]		GCA_019857665.1
*Engystomops pustulosus*	2.6	[97]		GCA_019512145.1
*Hymenochirus boettgeri*	3.2	[97]		GCA_019447015.1
*Limnodynastes dumerilii*	2.4	[98]		GCA_011038615.1
*Lithobates catesbeianus*	6.3	[58]		GCA_002284835.2
*Nanorana parkeri*	2.1	[36]		GCA_000935625.1
*Rana temporaria*	4.1	[99]		GCA_905171775.1
*Xenopus laevis*	2.7	[100]		GCA_017654675.1
*Xenopus tropicalis*	1.5	[101]		GCA_000004195.4
*Quasipaa spinosa*	2.6	[102]		https://datadryad.org/stash/landing/show?id=doi%3A10.5061%2Fdryad.ghx3ffbpw
*Rhinella marina*	2.6	[67]		http://gigadb.org/dataset/view/id/100483
*Spea multiplicata*	1.1	[103]		https://gsajournals.figshare.com/articles/dataset/Supplemental_Material_for_Seidl_et_al_2019/8303672?file=15558683
*Ranitomeya imitator*	6.8	[104]		GCA_905332335.1

### Repetitive element classification

We annotated the repetitive sequences using a de novo and homology-based method. First, we created a species-specific consensus sequence library using RepeatModeler v2.0.2 [77]. Next, we downloaded the Repbase [78] sequence library and the Dfam [79] dataset, merged the data generated by RepBase, Dfam, and RepeatModeler, and created the final repetitive sequence library. We then used RepeatMasker v4.1.2 [80] to identify the TEs and perform annotation. After that, we used linear regression analysis of annotations of the genome of the 14 species to estimate the level of association between the abundance of repetitive sequences and the genome size. To identify the tandem repeats, we used Tandem Repeats Finder v4.09 (default parameter) [81]. To further understand the relationship between simple sequence repeats (SSRs) and genome size, we used Krait [28] to identify SSRs in 14 anura genomes.

### Dynamics and diversity analysis of the active TE

The diversity of TE in each species was measured using the Simpson’s and Shannon diversity indices. In Simpson’s diversity index, D = $1-(\frac{\sum n(n-1)}{N(N-1)}$)[82], n represents the proportion of the genome occupied by each TE, and N represents the ratio of the sum of TEs to the size of the genome. In the Shannon diversity index $H$= $-\sum { p}_{i}{\text{ln}p}_{i}$ [83], ${p}_{i}$ is the proportion of sequences belonging to TEs type i. This is similar to the case in ecological community diversity assessment where ${p}_{i}$ represents the number of individuals belonging to the same species. In the equations above, TE diversity was assessed at the level of TE subclasses, namely LTRs, LINEs, SINEs, and DNA transposons, considered as species. Finally, we analyzed the relationship between genome size and diversity indices using linear regression equations [84].

To understand the dynamics and activity of TEs in each species, we used a Perl script parseRM.pl (parameter -l 50,1 -v) [85] to parse the output file (.fa.out) from RepeatMasker. We then calculated the percentage of divergence of TEs consensus sequences. As TE proliferation may influence genome size evolution, we used Kimura distance as a measure to evaluate the historical dynamics of TE expansion in 14 species to uncover the process of genome size variation. Finally, we used histograms to show the percentage of divergence of consensus sequences belonging to the same TEs types.

### Identification of the LTR-RTs

We implemented LTRharvest software [86] to identify intact LTR-RTs and analyze their impact on genome size and evolution. We run LTRharvest (parameter values -minlenltr 100 -maxlenltr 7000 -mintsd 4 -maxtsd 6 -motif TGCA -motifmis 1 -similar 85 -vic 10 -seed 20 -seqids yes) after creating the necessary files by Suffixerator (parameter values -tis -suf -lcp -des -ssp -sds -dna). The output file from LTRharvest was then inputted in LTR_retriever v 2.9.0 [87] using default settings for further analysis. As per the method described by Jukes et al. [88], we calculated the insertion time of LTR-RTs as T = k/2r, where k represents the divergence rate and a neutral mutation rate of 1.38 × 10^(-8) per site per year [89].

### Microsatellite sequence analysis

Given that compound SSRs are more complex and derived from the recombination of homologous SSRs [90], we only considered P-SSRs and I-SSRs in our study. To identify and classify SSRs in the genome of each species, we used Krait v 1.3.3 [28] with default settings. After that, we calculated the relative abundance (total SSRs/total valid length), density (total SSR length/total valid length), and percentage of sequences covered by SSRs. Using linear regression equations, we further determined the relationship between genome size and the aforementioned indices [84].

### Estimating the distribution of the non-coding regions of the genome

We used the methods of Francis and Worheide [47] to calculate the exonic and genic sequences in the genome of each species. We used a Python script (available at https://bitbucket.org/wrf/sequences/src/master/gtfstats.py) that takes genome and annotation files as input files. We then tallied the number of introns, exons, intronic gaps, intergenic regions, and intergenic gaps in each genome and estimated the average length of introns and exons. Finally, we employed linear regression [84] to evaluate the relationship between genome size and the number of exonic and genic sequences.

### Phylogenetic relationships and ancestral state reconstruction

We estimated the phylogenetic relationships between the 14 anuran species using OrthoFinder v 2.5.4 [91]. OrthoFinder assigned 522,457 genes, accounting for 93.2% of the total, to 27,105 orthogroups. Among the identified orthogroups, 5338 contained genes from all species, and 9 of these orthogroups consisted solely of single-copy genes. Protein-coding sequences were then used to infer the species trees, with analysis performed using the Species Tree inference from All Genes (STAG) method [92] utilizing the 5338 orthogroups identified using OrthoFinder.The resulting trees were visualized using iTOL [93]. To better understand the evolution of ancestral genome size, we reconstructed the ancestral state of genome size and TE proportion using the Maximum Likelihood approach and the fastAnc function in the R package phytools [94]. To evaluate the relationships between the species-specific genome size and TEs, we obtained the evolutionary branch information on the clade of each species using Lifemap [95] and combined this information with the ancestral state. We finally visualized the trees using iTOL.

### Evaluate the relationship between genome size and habitat

To understand the potential correlation between genome size and habitat diversity, we obtained habitat type data for each species from the IUCN archives (www.iucnredlist.org). Species with no detailed habitat type were removed in this analysis. We counted each species’ habitat type (Supplementary Materials 1: Table S6) and used a linear regression model [84] to assess the potential correlation between genome size and habitat diversity. In this analysis, habitat diversity is defined as the species’ niche width.

## Electronic supplementary material

Below is the link to the electronic supplementary material.

Supplementary Material 1

Supplementary Material 2

## Data Availability

The data and materials that support the findings of this study are available in the main text and supplementary material of this article.

## Abbreviations

TEs: Transposable Elements
LTRs: Long Terminal Repeat retrotransposons
LINEs: Long Interspersed Nuclear Elements
SINEs: Short Interspersed Nuclear Elements
SSRs: Simple Repeat Sequences
P-SSRs: Perfect SSRs
C-SSRs: Compound SSRs
I-SSRs: Compound SSRs
C-SSRs: Imperfect SSRs
MAY: million years ago
IUCN: Species Tree inference from All Genes
STAG: International Union for Conservation of Nature
BUSCO: Benchmarking Universal Single-Copy Orthologs
