# Supplementary material for: Comparative genomics reveals insights into anuran genome size evolution
Source: BMC Genomics. 2023 Jul 6;24:379. doi: 10.1186/s12864-023-09499-8 (PMC10324214; doi:10.1186/s12864-023-09499-8)
Supplement: Supplementary file 2 — Supplementary Material 2 [file 12864_2023_9499_MOESM2_ESM.docx]

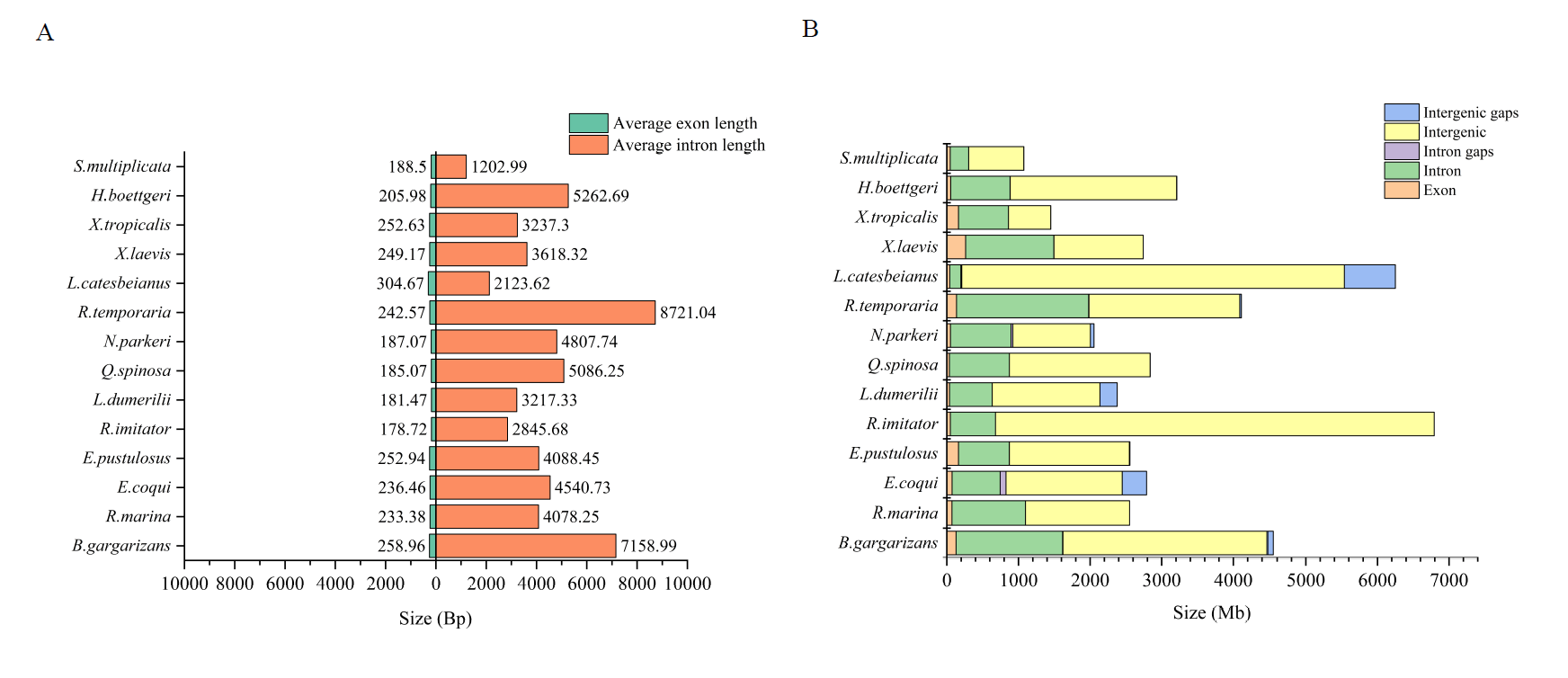


**Fig. S1 shows the landscape of non-coding regions in 14 species.** Figure A shows the average length of introns and exons, while figure B shows the length of introns, exons, and intergenic regions. In Figure A, the orange bars represent the average intron length, and the green bars represent the average exon length in different species. In Figure B, the blue bar represents the length of the intergenic gaps, yellow represents the intergenic length, purple represents the length of the intronic gaps, green represents the intron length, and orange represents the exon length.


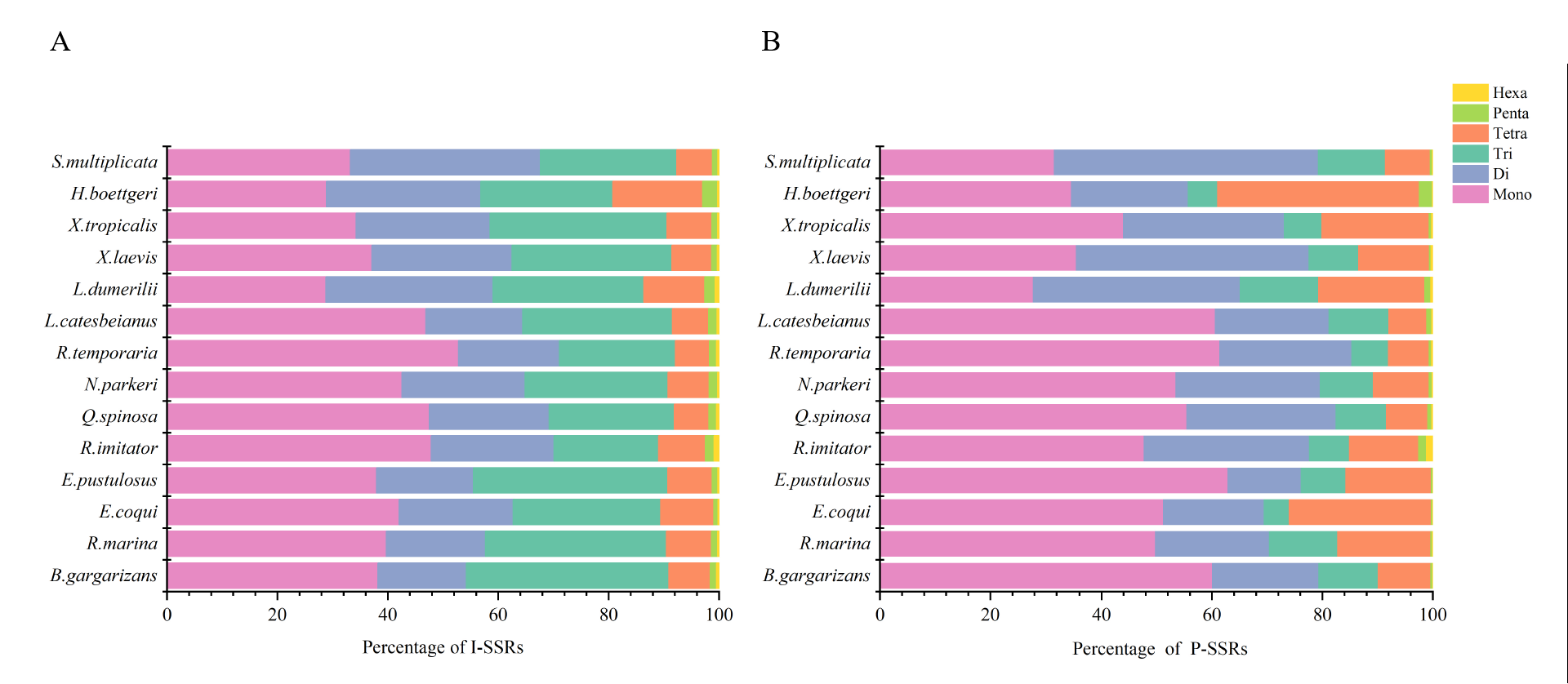


**Fig. S2 shows the percentage of six categories of SSRs in the 14 anuran genomes.** Figures A and B indicate the percentage of I-SSRs and P-SSRs in the 14 anuran genomes, respectively. In the figure, fuchsia, indigo, green, orange, yellow-green, and yellow represent the six SSRs types of Mononucleotide, Dinucleotide, Trinucleotide, Tetranucleotide, Pentanucleotide, and Hexanucleotide, respectively.


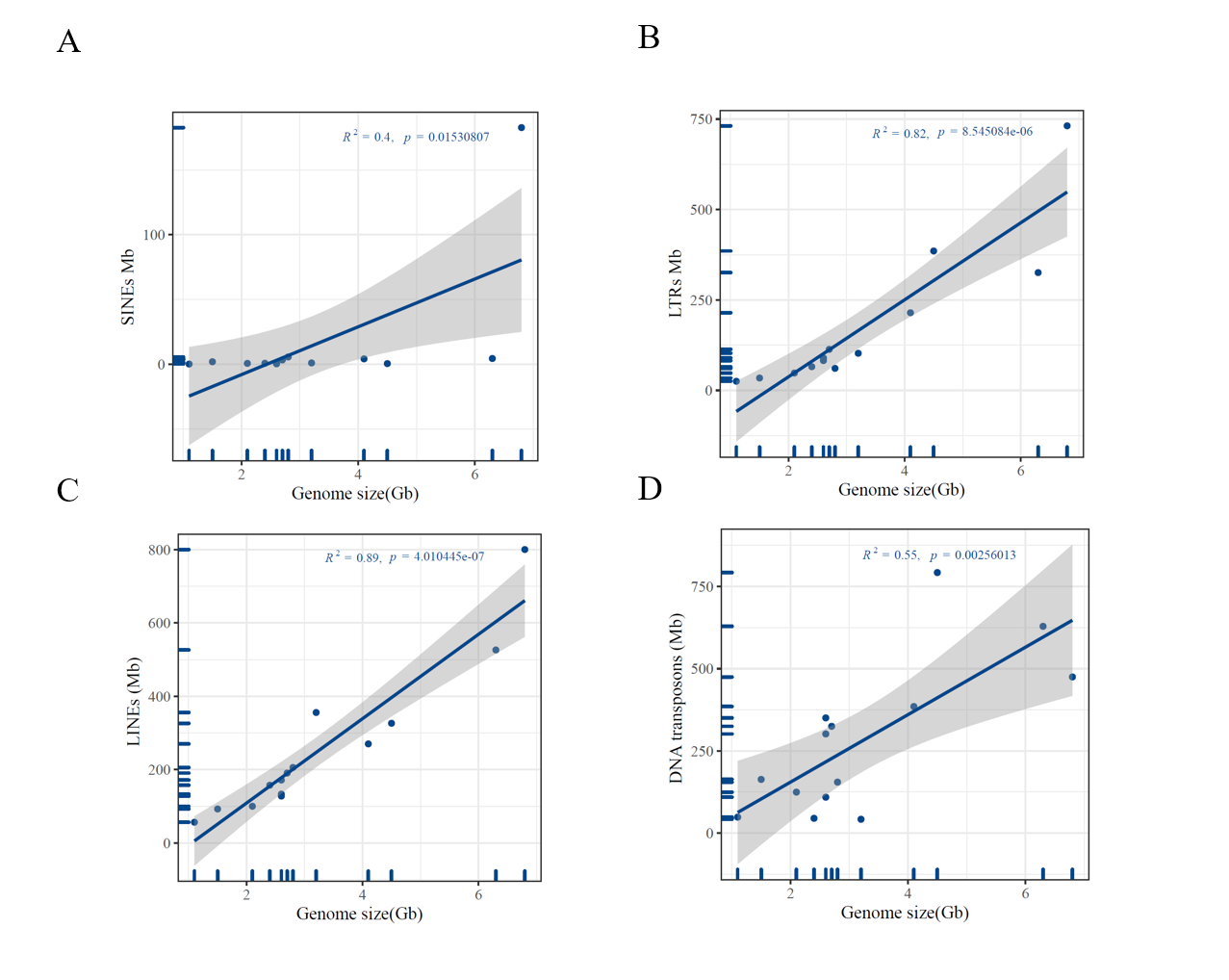


**Fig. S3 shows the linear model regressions of transposable elements with genome size variations among 14 anuran species** (gray shade represents the 95% confidence interval). Figures A, B, C, and D indicate the associations between the abundance of SINEs, LTRs, LINEs, DNA transposons, and genome size, respectively.


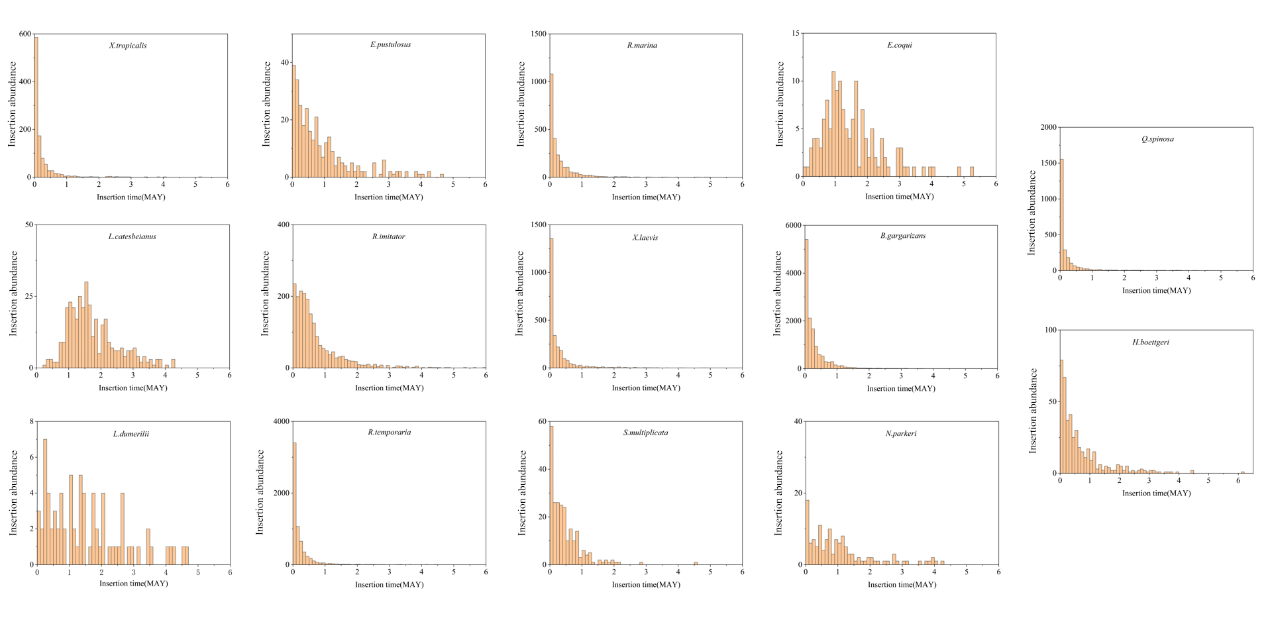


**Fig. S4 Insertion time and insertion abundance distribution of intact LTR-RTs in 14 anuran genomes.** The X and Y axes of the figure indicate the insertion time (million years) and insertion abundance of intact LTR-RTs, respectively.


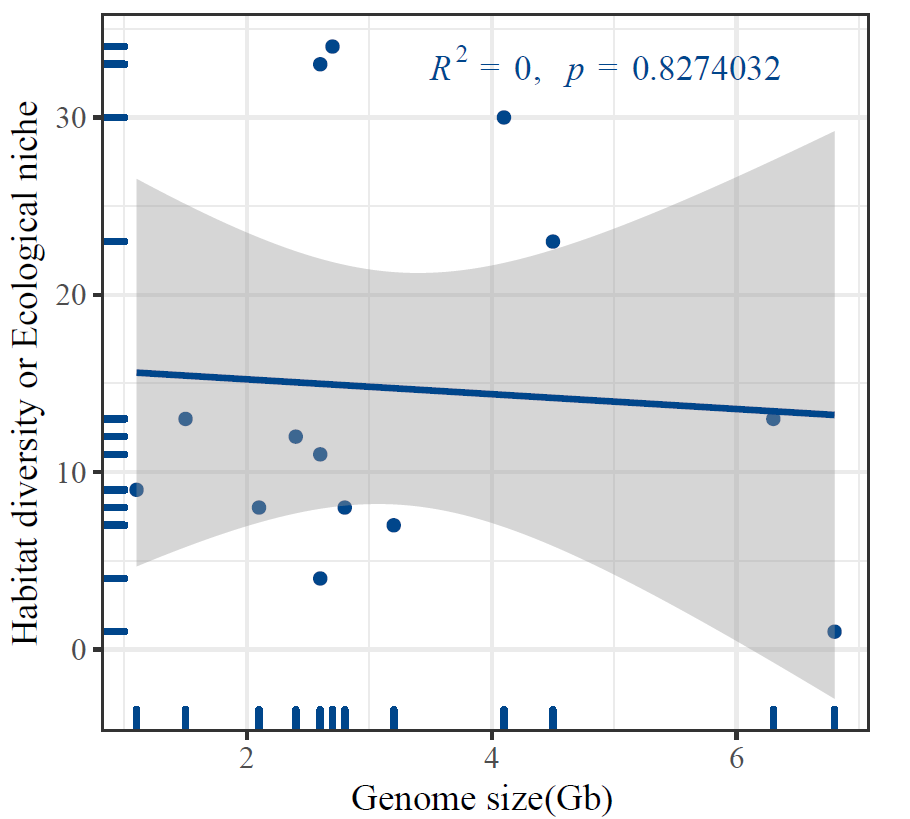


**Fig. S5 Association between genome size and habitat diversity or ecological niche width in 14 anuran genomes.** (Gray shade represents the 95% confidence interval). The X-axis indicates genome size and the Y-axis indicates habitat diversity or ecological niche width.


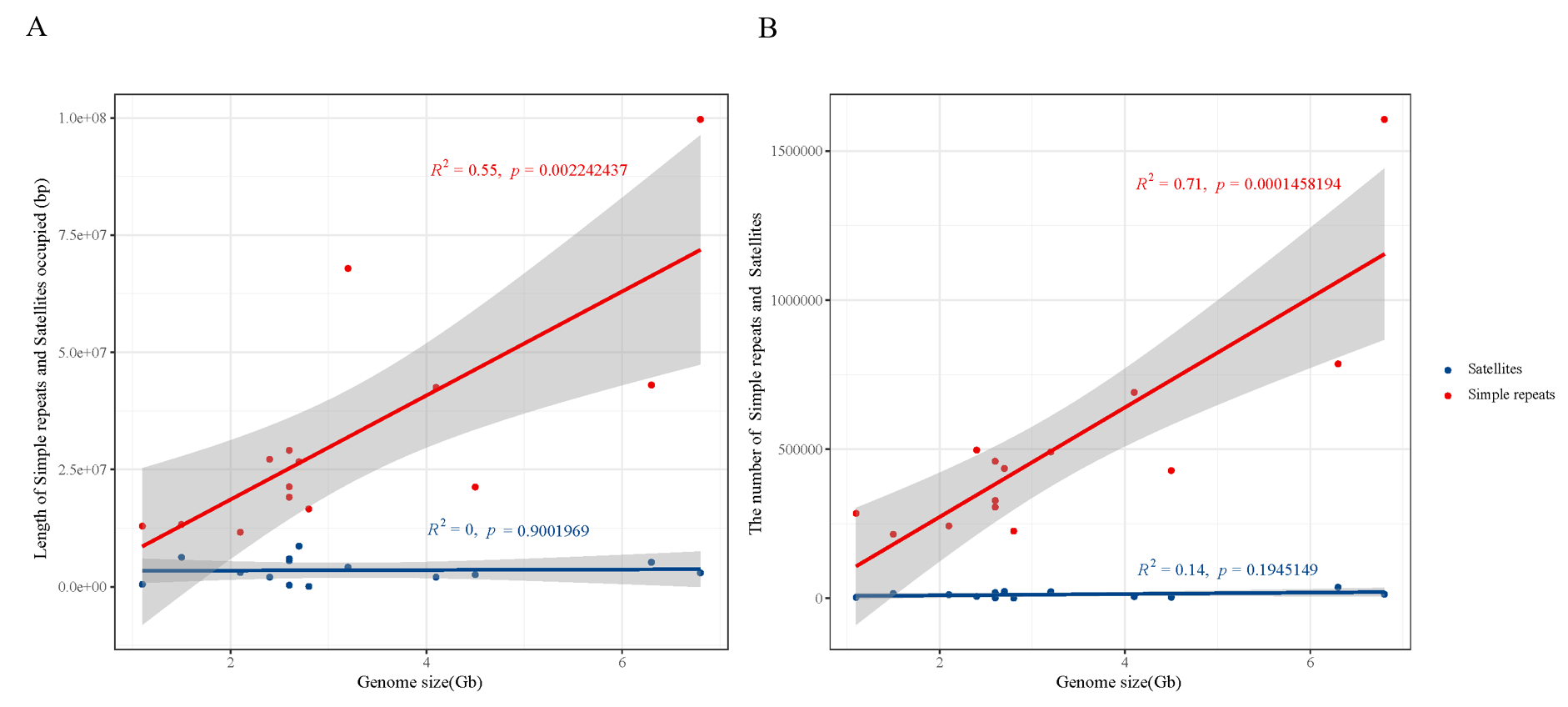


**Fig. S6 The relationship between genome size and tandem repeats.** The figure on the left shows the relationship between genome size and length of satellite and simple repeat sequences, respectively. On the right is the association of genome size with the number of satellite sequences and simple repeats, respectively. (Gray shade represents the 95% confidence interval). The x-axis represents the genome size, while the y-axis represents the length (left) and number (right) of elements in the genome, respectively.


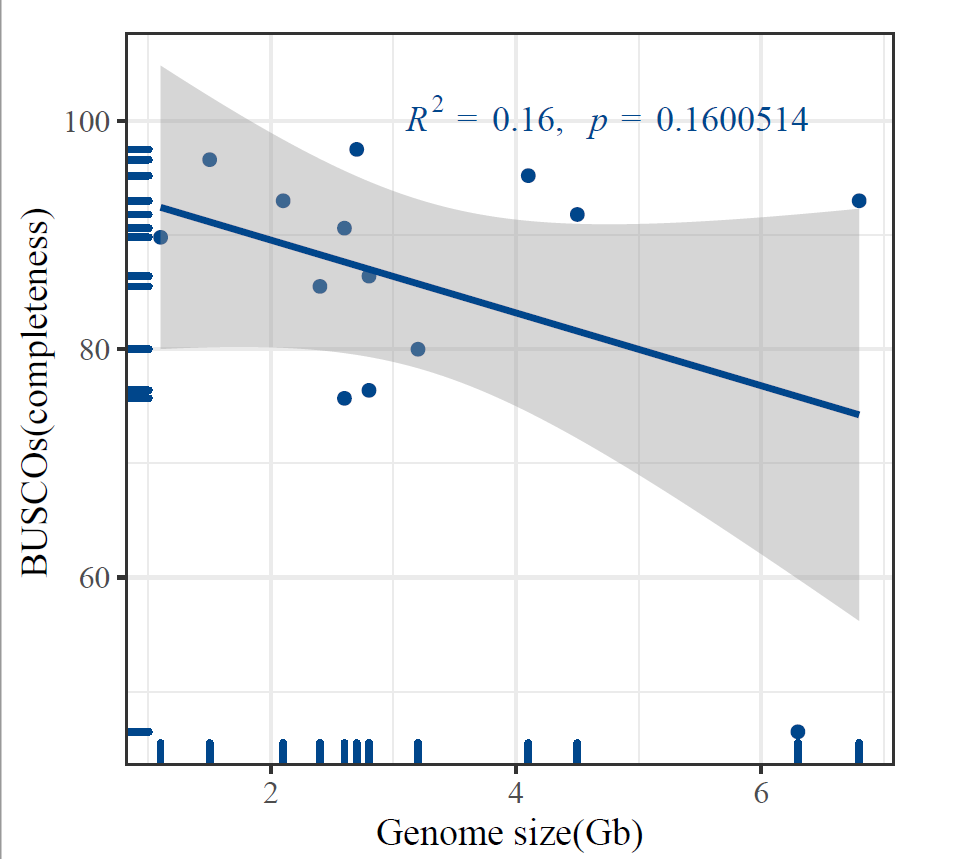


**Fig. S7 The relationship between genome size and genome quality.** The figure represents the relationship between genome size and genome quality completeness for 14 species. (Gray shade represents the 95% confidence interval). The X-axis represents genome size, and the Y-axis represents the evaluation quality of BUSCO.


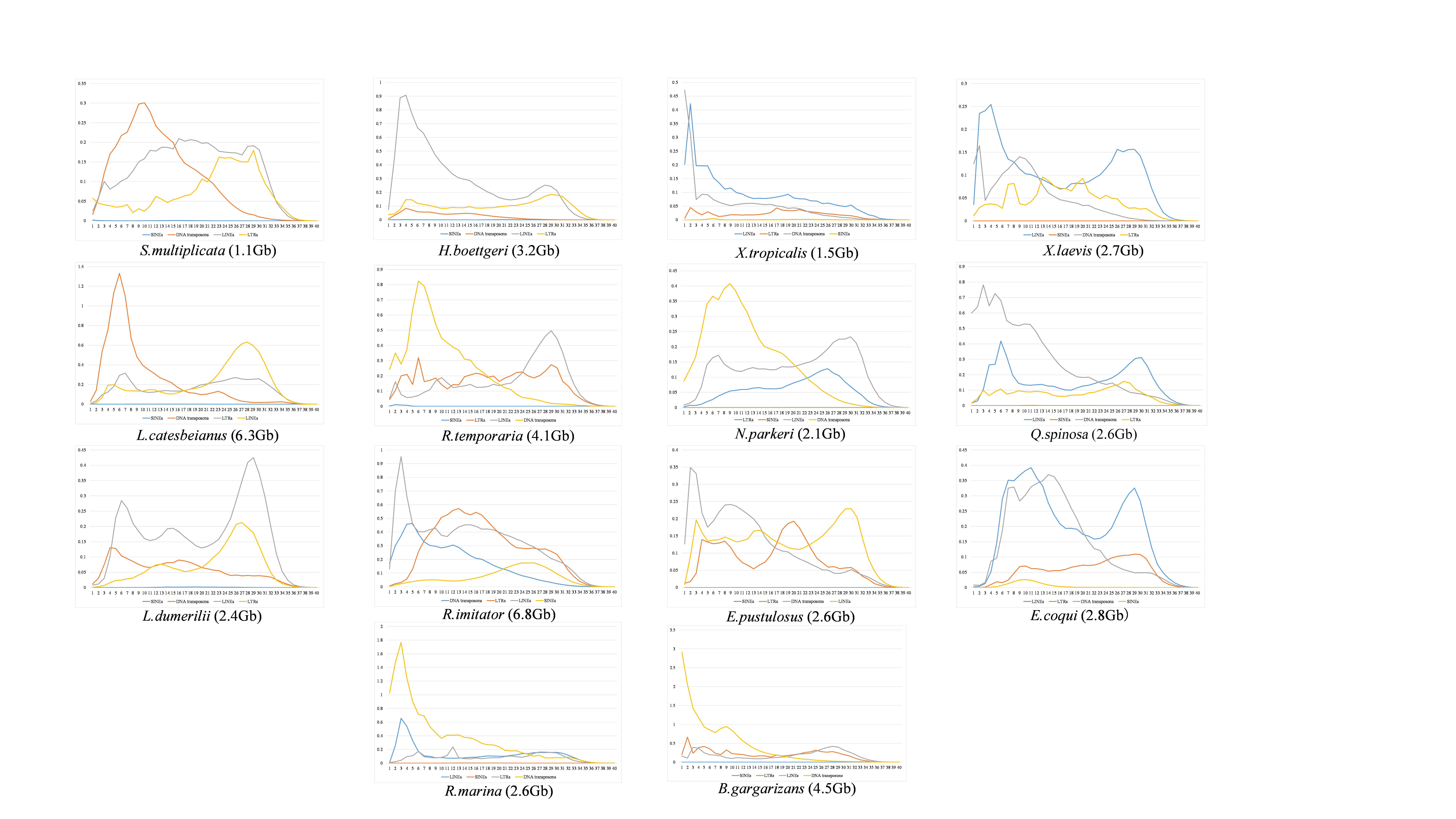


**Fig. S8 Age distribution curve of transposable elements in Anuran genomes .** The Y-axis shows the genomic coverage of different types of TEs, and the X-axis shows the Kimura substitution level as a percentage from 0 to 40. The Y-axis represents TE abundance as a proportion of the genome (e.g., 1.0 = 1% of the genome).
